# Association of systemic immune-inflammatory index with stress urinary incontinence in U.S. Adults: NHANES 2007–2016

**DOI:** 10.1371/journal.pone.0353080

**Published:** 2026-07-09

**Authors:** Sijia Ma, Linlin Qian, Yuhua Jiang, Meng Wang, Shijie Xu, Chao Wang

**Affiliations:** 1 Department of Nephrology, The Third Affiliated Hospital of Zhejiang Chinese Medical University (Zhongshan Hospital of Zhejiang Province), Hangzhou, Zhejiang, China; 2 Institute of Basic Theory of Traditional Chinese Medicine, China Academy of Chinese Medical Sciences, Beijing, China; The First Affiliated Hospital of Soochow University, CHINA

## Abstract

**Background:**

The aim of this study is to explore the association between the systemic immune-inflammatory index (SII) and stress urinary incontinence (SUI).

**Methods:**

We used information obtained from the National Health and Nutrition Examination Survey (NHANES) that was conducted between 2007 and 2016. The weighted multivariate logistic regression model was used in order to evaluate the relationship that exists between the SII index and the SUI measurement. In addition, a technique from the field of smooth curve fitting was used to investigate the linear connection that exists between these variables. In order to determine whether or not the SII index and SUI relationship remained stable across a variety of demographic strata, subgroup analyses were carried out.

**Results:**

The research had a grand total of 20,849 individuals. After full adjustment for confounders, each 1-unit increase in log₁₀(SII) was associated with a 29.7% increase in the odds of SUI (OR=1.297, 95%CI 1.028–1.636, P = 0.032). Categorizing the SII index into quartiles revealed that even in the highest SII quartile, there remained a significant positive association with SUI when compared to the lowest quartile (OR =1.177, 95%CI 1.013–1.368, *P* = 0.038). Furthermore, an augmented correlation between alcohol consumption and SUI was detected among drinkers compared to non-drinkers (*P* < 0.05).

**Conclusions:**

Higher systemic inflammatory burden reflected by SII is statistically significantly associated with increased odds of SUI in the U.S. adult population, though the effect size is small; this association is particularly prominent in alcohol drinkers.

## Introduction

Urinary incontinence (UI) is a common condition affecting a large portion of the population, often leading to significant decline in patients’ quality of life and social participation. It imposes a considerable psychological burden on affected individuals and increases social and economic pressures [1].

Stress urinary incontinence (SUI) is the most prevalent subtype of UI, characterized by involuntary urine leakage triggered by activities that increase intra-abdominal pressure (e.g., sneezing, coughing, laughing, or physical exertion), resulting from impaired urethral continence mechanisms [2]. According to statistical data, SUI significantly impacts 10% ~ 40% of women [3]. Additionally, during 2017–2020, over one-third of men aged 60 years and older reported experiencing any form of urinary incontinence, with urgent urinary incontinence being the most common (31.3%) [4]. Currently, the management of SUI mainly includes lifestyle modifications, physical therapy, pharmacological interventions, and surgical approaches. These modalities aim to alleviate symptoms and enhance the quality of life for affected individuals to varying degrees [5].

The systemic immune inflammation index (SII), calculated by multiplying platelet count by neutrophil-to-lymphocyte ratio, has been recognized as predictive of inflammatory status in humans [6]. Elevated SII typically reflects increased chronic inflammation and has been identified as a prognostic marker for various diseases, including cancer, cerebral hemorrhage, and coronary artery stenosis [7–9]. Chronic inflammation is also associated with degenerative changes in the pelvic floor support structures (e.g., muscles, fascia, ligaments), which may compromise their supportive capacity [10]. However, the relationship between SII and SUI remains unclear.

To address this research gap, our study used a nationally representative sample of U.S. adults aged ≥20 years to explore the association between SII and SUI. Early identification, diagnosis and intervention may help reduce the incidence of SUI and its complications, as well as alleviate patient distress.

## Methods

### Study population

NHANES is a nationally representative survey designed to assess the health status of the U.S. population. Over two decades, this program has collected data on demographics, examinations, nutrition, and laboratory findings. NHANES employs a complex, multi-stage stratified random sampling method to ensure the sample is representative of non-institutionalized U.S. citizens. All participants provided written informed consent. For detailed study protocols, refer to the official website (https://www.cdc.gov/nchs/nhanes).

**Fig 1:**
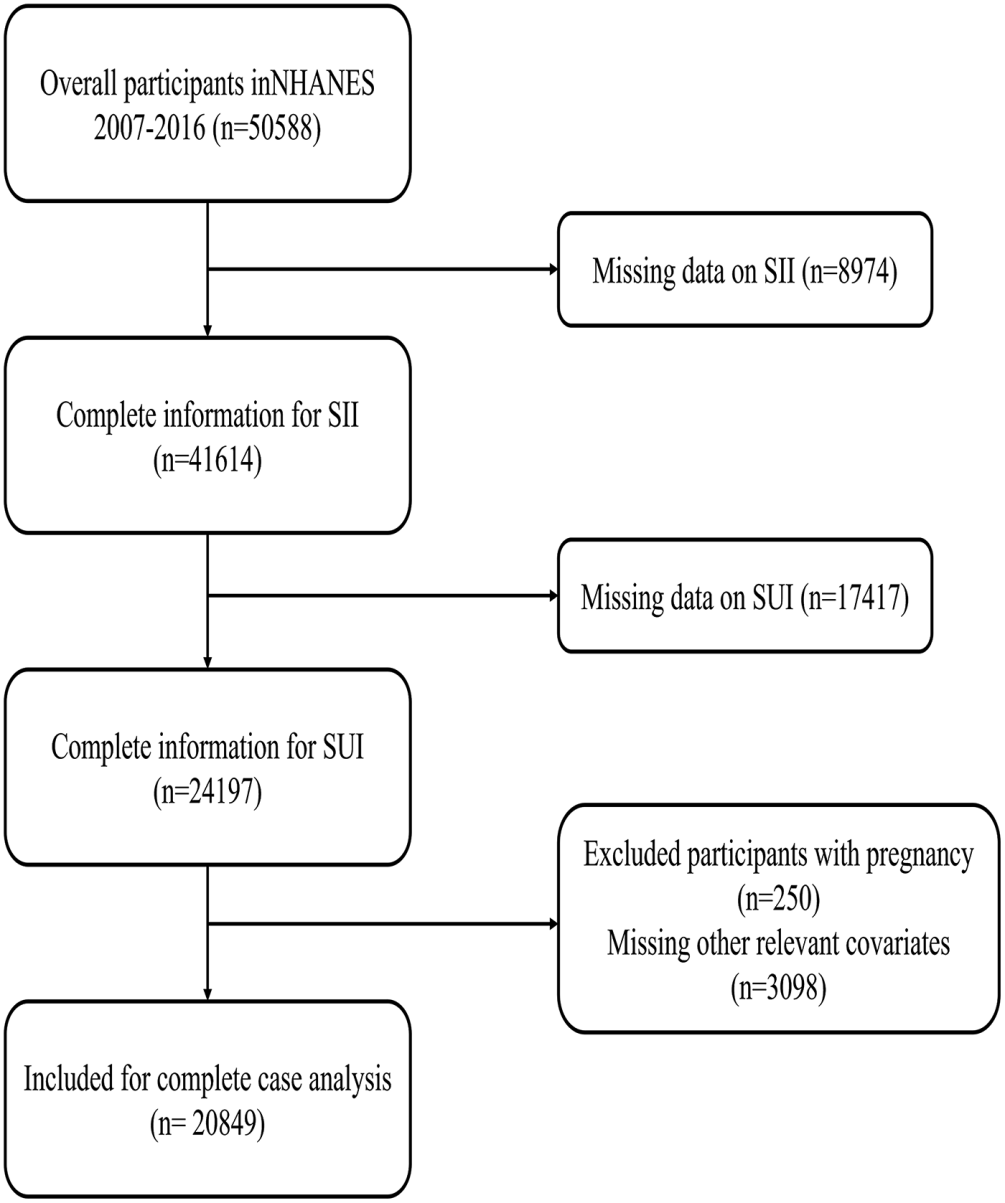
Participant selection criteria.

In this study, we analyzed data from five NHANES survey cycles (2007–2016). Initially, 50,588 participants were included. We excluded individuals with missing SII data (n = 8974), missing SUI data (n = 17417), pregnant participants (n = 250), and those with missing information on other relevant covariates (n = 3098). Finally, 20,849 participants were included in the analysis (Fig 1).

### Assessment of stress urinary incontinence

SUI was assessed using a self-reported questionnaire focusing on symptoms occurring in the past 12 months. Participants were asked: “Have you experienced involuntary leakage or loss of even a small amount of urine during activities such as coughing, lifting, or exercising?” [11–13]. A positive response was defined as having SUI.

### Assessment of systemic immune-inflammation index

Complete blood counts were performed using a Coulter® DxH 800 analyzer under the guidance of trained medical professionals, with results reported in ×10⁹ cells/µl. The SII was calculated as follows [14,15].


$$\begin{array}{c} SII=\frac{\text{ platelet count}\times \text{neutrophil count}}{\text{lymphocyte count}} \end{array}$$

### Covariates of interest

Covariates included demographic and lifestyle factors such as gender, age, race/ethnicity, educational attainment, marital status, family poverty-to-income ratio (PIR), body mass index (BMI), waist circumference, smoking habits, alcohol consumption, level of physical activity (vigorous/moderate), as well as the presence of status diabetes, hypertension, and high cholesterol. Smoking status was categorized into three groups: never smoked (lifetime consumption <100 cigarettes), former smokers (lifetime consumption >100 cigarettes but currently not smoking), and current smokers (lifetime consumption ≥100 cigarettes and currently smoking) [12,13]. Diabetes status was categorized as yes, no, or borderline.

### Statistical analysis

To account for NHANES’ complex multi-stage sampling design, we incorporated sample weights, SDMVPSU (SDMV Primary Sampling Unit), and SDMVSTRA (stratification) into statistical analyses to ensure representativeness of the non-institutionalized U.S. civilian population. Continuous data were presented as weighted means ± standard errors (SE), and categorical variables as weighted proportions. Baseline characteristics across SII quartiles were compared using survey-weighted chi-square tests (categorical variables) and survey-weighted linear regression (continuous variables).

Subgroup analysis was performed to explore the stability of the SII-SUI association across different strata. Smooth curve fitting was used to evaluate the nonlinear relationship between SII and SUI, and a threshold effect analysis model was employed to identify potential inflection points. The two-sided *P*-value of less than 0.05 was used to evaluate statistical significance. R 4.2.2 and EmpowerStats 4.2 were the software programs used for the statistical studies.

## Results

### Participant characteristics

The study included 20,849 participants (10,222 men and 10,627 women) with a mean age of 51.44 ± 17.35 years. The prevalence of SUI in the cohort was 23.67%. After log₁₀ transformation, the SII quartiles were: Q1 (0.18 ~ 2.51), Q2 (2.51 ~ 2.66), Q3 (2.66 ~ 2.81), and Q4 (2.81 ~ 4.45). Compared with participants in the lowest SII quartile (Q1), those in the highest quartile (Q4) were older, more likely to be non-Hispanic white women, had lower household income and educational attainment, and higher rates of living alone, smoking, elevated BMI and waist circumference, hypertension, hyperlipidemia, diabetes, and SUI (all P < 0.001; Table 1).

**Table 1:** Baseline characteristics of participants by the SII index quartiles, weighted.

Characteristic	Total	Q1, N = 5211	Q2, N = 5213	Q3, N = 5212	Q4, N = 5213	p-value
Age	51.44 ± 17.35	48.03 ± 0.38	48.30 ± 0.33	49.34 ± 0.35	50.96 ± 0.38	<0.001
Sex						<0.001
Male	49.03	53.67	50.74	49.26	43.22	
Female	50.97	46.33	47.70	52.30	56.79	
Race/ethnicity (%)						<0.001
Mexican American	13.90	7.39	7.56	8.05	7.32	
Other Hispanic	10.49	5.25	5.48	5.55	4.88	
Non-Hispanic White	44.20	61.87	69.65	71.61	75.21	
Non-Hispanic Black	20.67	18.10	9.45	8.01	6.87	
Other Race- Including Multi-Racial	10.74	7.39	7.86	6.79	5.73	
Education level (%)						<0.001
Less than high school	23.07	16.06	13.73	14.84	15.72	
High school or GED	22.17	21.18	20.12	21.61	22.96	
Above high school	54.76	62.75	66.13	63.55	61.33	
Marital status (%)						<0.001
Living alone	39.70	35.03	32.21	35.27	38.60	
Married or living with partner	60.30	64.98	67.79	64.73	61.41	
Family PIR (%)	2.59 ± 1.64	3.02	3.09	3.08	3.00	0.126
Smoking status (%)						<0.001
Current smokers	19.05	17.00	17.30	18.33	21.00	
Former smokers	25.67	24.69	25.45	26.04	27.83	
Nonsmokers	55.28	58.31	57.26	55.63	51.17	
Alcohol intaking (%)						0.228
Drinkers	71.22	76.06	77.44	78.16	76.89	
Nondrinkers	28.78	23.94	22.56	21.84	23.11	
BMI	29.43 ± 6.96	28.24 ± 0.12	28.66 ± 0.16	29.53 ± 0.14	30.25 ± 0.16	<0.001
Waist circumference	100.16 ± 16.09	97.37 ± 0.33	98.84 ± 0.35	100.83 ± 0.33	102.28 ± 0.32	<0.001
Hypertension (%)						<0.001
Yes	40.07	32.30	33.32	35.20	39.80	
No	59.93	67.70	66.68	64.80	60.20	
High cholesterol (%)						<0.001
Yes	39.06	34.17	37.60	38.67	39.39	
No	60.94	65.83	62.40	61.33	60.62	
Diabetes (%)						<0.001
Yes	14.52	9.12	9.34	10.85	13.16	
No	83.00	88.39	88.43	86.98	84.53	
Borderline	2.48	2.49	2.23	2.170	2.31	
Vigorous activity (%)						0.739
Yes	35.15	40.36	39.97	40.16	39.06	
No	64.85	59.64	60.03	59.84	60.94	
Moderate activity (%)						0.151
Yes	18.06	21.17	20.23	20.73	18.90	
No	81.94	78.83	79.77	79.27	81.10	
SUI						<0.001
Yes	23.67	19.10	23.40	25.83	27.53	
No	76.33	80.90	76.60	74.17	72.47	

### Relationship between SII index and SUI

Weighted multivariate logistic regression models were used to assess the association between SII index and SUI under three adjustment scenarios: crude (Model 1), minimally adjusted (Model 2), and fully adjusted (Model 3). Results showed a positive correlation between elevated SII and increased SUI risk.

When SII was treated as a continuous variable, after full adjustment for all covariates (Model 3), each 1-unit increase in log₁₀(SII) was associated with a significant increase in the odds of SUI (OR=1.297, 95% CI 1.028–1.636, *P* = 0.032). In the highest SII quartile(Q4), compared with Q1, the SUI risk increased by 17.7% after full adjustment (OR=1.177, 95% CI 1.013–1.368, *P* < 0.05; Table 2).

**Table 2:** Following the adjustment of various covariates, the SII index and the weighted association between SUI.

SUI	OR (95% CI), *P*‑value		
	**Model 1**	**Model 2**	**Model 3**
Log_10_(SII)	2.108 (1.719,2.584), 0.001	1.569 (1.243,1.980), 0.001	1.297 (1.028,1.636), 0.032
Quartiles			
Q1	Reference	Reference	Reference
Q2	1.294 (1.144,1.463), 0.001	1.266 (1.114,1.438), 0.006	1.218 (1.069,1.388), 0.004
Q3	1.475 (1.301,1.672), < 0.001	1.361 (1.182,1.566), < 0.001	1.277 (1.107,1.474), 0.001
Q4	1.601 (1.402,1.847), < 0.001	1.321(1.137,1.536), 0.005	1.177 (1.013,1.368), 0.038
***P* for trend**	< 0.001	0.003	0.032

OR: odds ratio, 95% CI: 95% confidence interval.

Model 1: Covariates without any adjustments.

Model 2: Adjusted for fundamental demographic variables: gender, age, and racial/ethnic

Model 3: Adjusted for all covariates: gender, age, race/ethnicity, education, marital status, family PIR, smoking status, alcohol use, degree of activity, BMI, waist circumference, diabetes, hypertension, and high cholesterol.

**Fig 2:**
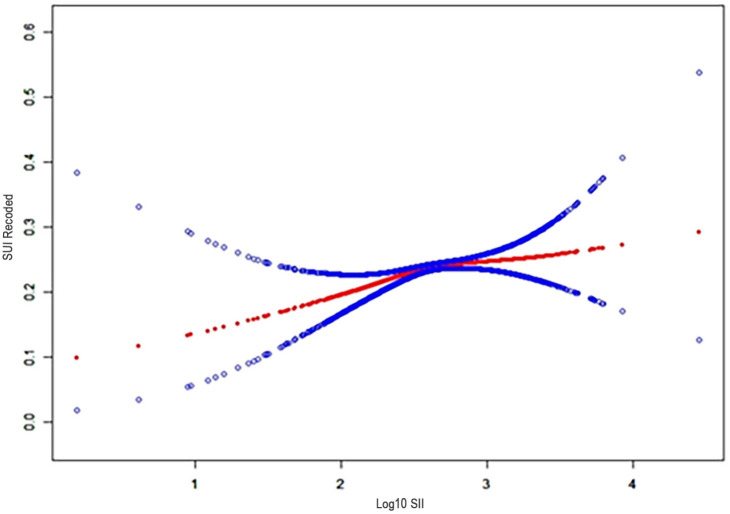
Smooth curve fitting plot of the association between SII and SUI. The area between the two blue dashed lines represents the 95% CI. The red dashed line indicates a linear positive correlation between the SII index and SUI.

Furthermore, smooth curve fitting analysis indicated a linear and positive correlation between the SII index and SUI (Fig 2).

### Threshold effect analysis

A two-piecewise linear regression model was used to identify the threshold effect between log₁₀SII and SUI (Table 3). The results showed that the prevalence of SUI peaked when log₁₀SII reached 2.604. Below this breakpoint (log₁₀SII < 2.604), there was a significant positive correlation (OR=1.646, 95%CI: 1.152–2.351, *P* < 0.05). Above the threshold (log₁₀SII > 2.604), the positive correlation persisted but was not statistically significant (OR=1.038, 95%CI: 0.803–1.343, *P* > 0.05).

**Table 3:** The two-piecewise linear regression model analysis between log_10_SII and SUI threshold effect.

	Adjusted OR (95% CI), *P*-Value
**Fitting by standard linear model**	
OR	1.241 (1.056,1.459), 0.009
**Fitting by two-piecewise linear model**	
Breakpoint (K)	2.604
OR1 (< 2.604)	1.646 (1.152,2.351), 0.006
OR2 (> 2.604)	1.038 (0.803,1.343), 0.775
OR2/OR1	0.631 (0.376,1.057), 0.081
Logarithmic likelihood ratio test *P*-Value	0.079

### Subgroup analysis

**Fig 3:**
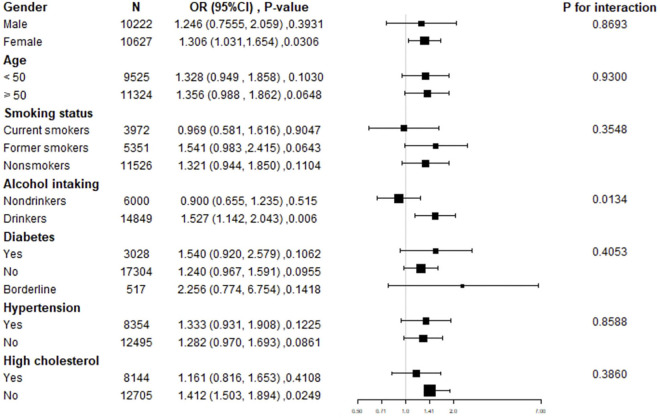
Subgroup analysis of the association between SII index and SUI. Squares represent OR values (ratio of SUI occurrence between groups), and horizontal lines represent 95% CI.

A subgroup study was conducted to assess the stability of the connection between the SII index and SUI across various levels. The stratification criteria were gender, age, smoking status, diabetes, hypertension, and high cholesterol. Fig 3 demonstrates that alcohol use might potentially affect the relationship between the SII index and SUI. When comparing drinkers to nondrinkers, the SII index showed a stronger association with SUI (*P* for interaction < 0.05).

## Discussion

This study used a large, nationally representative sample from the 2007–2016 NHANES database to evaluate the association between SII and SUI in U.S. adults. Among 20,849 participants, we found that elevated SII was positively associated with SUI. Quartile analysis confirmed a dose-dependent relationship, and smooth curve fitting revealed a linear association (log10SII inflection point: K < 2.604, *P* < 0.05). Notably, the association was stronger in alcohol consumers, suggesting that lifestyle factors may modify this relationship. This enhanced association may be attributed to several potential pathways: first, alcohol itself exerts pro-inflammatory effects that could amplify systemic inflammatory burden; second, alcohol consumption may be associated with weight gain, increased coughing, or other behaviors that exacerbate urinary leakage; third, alcohol use might serve as a surrogate for unmeasured lifestyle factors (e.g., poor dietary habits) that contribute to both inflammation and SUI. However, we acknowledge that this interaction finding should be interpreted cautiously, given the multiple subgroup tests conducted, which may increase the risk of false positivity.

These findings position the SII—a well-validated marker of systemic inflammation [9,10,16–18]—as a promising predictor for SUI risk, highlighting its potential value in clinical assessment. SUI is a prevalent condition that significantly impacts patients’ psychological health and societal functioning [19]. Its primary pathological mechanisms include impaired neural control dysfunction of urinary tract structures, compromised pelvic floor support, and dysfunctional urethral sphincter, leading to inability to maintain urethral closure during increased intra-abdominal pressure [20,21]. Gender-specific risk factors exist: in women, pregnancy and vaginal delivery can cause urethral sphincter injury; in men, SUI often results from neurovascular bundle and fascia trauma during radical prostatectomy [22–24]. Additional risk factors such as smoking, obesity, and constipation further exacerbate the condition [25,26]. SUI is also associated with comorbidities including sleep disturbances, depression, insulin resistance, and urinary tract infections [27–30]. Despite these well-documented risk factors and mechanisms, the relationship between SII and SUI remains unexplored. Given the potential mediating role of chronic inflammation in pelvic floor dysfunction, the present study focuses on the value of SII as a novel predictive marker for SUI, aiming to provide critical evidence for the early identification, prevention, and clinical management of SUI, thereby filling the existing research gap in this field.

Notably, our results align with emerging evidence linking inflammation to pelvic floor dysfunction (PFD). PFD is a common urological condition encompassing pelvic organ prolapse (POP), SUI, fecal incontinence, and overactive bladder syndrome (OAB) [31]. Recent studies have increasingly implicated immune-inflammatory responses in the pathogenesis of OAB, and several studies have reported a positive correlation between SII levels and OAB [32,33].

The mechanistic plausibility of this association is supported by prior research. A systematic review of molecular processes in SUI (in humans and animals) found that SUI is related to changes in extracellular matrix metabolism, apoptosis, and inflammation [34]. Animal studies have shown lower expression of smooth muscle alpha-2 actin (ACTa2) in SUI models compared to controls [35], while human studies reported decreased transforming growth factor-beta 1 (TGF-β1) protein levels in SUI patients [36]. Another animal study showed increased protein expression of TNF-α, Interleukin-1 (IL-1) in the SUI model compared to controls [37]. A serum proteomic analysis of SUI patients (n = 19) and healthy controls (n = 19) identified 33 proteins associated with inflammatory responses, coagulation reactions, cellular stress, and cytoskeletal stability/mobility in SUI samples [38].

Several mechanisms may explain the positive correlation between SII and SUI. As previously reported, elevated SII is associated with increased levels of inflammatory markers such as C-reactive protein (CRP), TNF-α, and IL-6 [39,40]. Inflammation plays a significant role in SUI pathogenesis by damaging pelvic floor support structures and impairing urethral sphincter function [41–43]. Research indicates that inflammation can lead to structural and functional abnormalities in pelvic floor tissues, including alterations in muscle contraction strength and neural regulation, thereby increasing the risk of urinary incontinence [44,45]. Additionally, inflammatory processes may be associated with mucosal inflammation of the urethra and bladder, which could affect urine storage and voiding, potentially worsening SUI symptoms [46,47]. Moreover, allergies, urinary tract infections, and gynecological inflammation have been shown to worsen SUI symptoms [48,49]. Collectively, these findings suggest that SII may serve as a potential therapeutic target for SUI.

This study aimed to determine the association between SII and SUI in U.S. adults. Rigorous sampling design and weighting methods were used to ensure statistical analyses accurately reflected the demographics of the U.S. adult population. Nevertheless, several limitations warrant acknowledgment.

Primarily, our study adopted a cross-sectional design, precluding the exploration of a causal relationship between the SII index and SUI. Additionally, constraints inherent to the SUI questionnaire design within the NHANES database necessitated reliance on participant self-reports regarding symptoms and medical history, potentially resulting in an underestimation of SUI prevalence. This reliance on self-reporting mechanisms introduces variability influenced by participants’ interpretation of questions and varying educational backgrounds, thereby heightening concerns regarding recall bias and misclassification bias, particularly concerning the accurate differentiation between Urge Urinary Incontinence (UUI) and SUI. Furthermore, it’s imperative to acknowledge that the NHANES database exclusively encompasses data from the U.S. population. Consequently, future research endeavors should encompass prospective studies incorporating sizable cohorts from diverse global regions to validate the observed association between the SII index and SUI across different national demographics.

## Conclusion

To our knowledge, this study represents the inaugural examination of the correlation between the SII index and SUI within the US adult demographic. We found a statistically significant positive correlation, with higher SII index values linked to an increased risk of SUI, consistent across SII quartiles. Elevated SII may increase the risk of SUI by impairing pelvic floor structure and function through multiple mechanisms, including chronic inflammation, oxidative stress, metabolic disorders, and vascular dysfunction. These findings underscore the significant association between SUI and SII, highlighting the potential role of SII in urinary dysfunction. We thus advocate for clinicians to incorporate inflammatory evaluation into the comprehensive management of patients with urinary symptoms. Nonetheless, further investigations conducted on a global scale are imperative to corroborate and extend the generalizability of our observed associations.

## References

[1] Harland N, Walz S, Eberli D, Schmid FA, Aicher WK, Stenzl A, et al. Stress urinary incontinence: an unsolved clinical challenge. Biomedicines. 2023;11(9). doi: 10.3390/biomedicines11092486 37760927 PMC10525672

[2] Wu Y, Li P, Shi J, Li J, Zhang Y, Xiao B. Research trends of acupuncture therapy on stress urinary incontinence from 1992 to 2022: a bibliometric analysis. Heliyon. 2023;9(9):e19732. doi: 10.1016/j.heliyon.2023.e19732 37810054 PMC10559016

[3] Xu M, Zhou H, Pan Y, Xu Z, Liu X. Serum albumin levels and stress urinary incontinence in females: a retrospective study based on NHANES 2007-2016. Heliyon. 2023;9(11):e21757. doi: 10.1016/j.heliyon.2023.e21757 38027892 PMC10656249

[4] Cao C, Zhang C, Sriskandarajah C, Xu T, Gotto G, Sutcliffe S. Trends and racial disparities in the prevalence of urinary incontinence among men in the USA, 2001-2020. Eur Urol Focus. 2022;8(6):1758–67. doi: 10.1016/j.euf.2022.04.015 35562253

[5] Padilla-Fernández B, Ramírez-Castillo GM, Hernández-Hernández D, Castro-Díaz DM. Urodynamics before stress urinary incontinence surgery in modern functional urology. Eur Urol Focus. 2019;5(3):319–21. doi: 10.1016/j.euf.2019.03.018 30987929

[6] Di X, Liu S, Xiang L, Jin X. Association between the systemic immune-inflammation index and kidney stone: a cross-sectional study of NHANES 2007-2018. Front Immunol. 2023;14:1116224. doi: 10.3389/fimmu.2023.1116224 36895572 PMC9989007

[7] Xie R, Xiao M, Li L, Ma N, Liu M, Huang X, et al. Association between SII and hepatic steatosis and liver fibrosis: a population-based study. Front Immunol. 2022;13:925690. doi: 10.3389/fimmu.2022.925690 36189280 PMC9520084

[8] Albany C. Systemic immune-inflammation index in germ-cell tumours: search for a biological prognostic biomarker. Br J Cancer. 2018;118(6):761–2. doi: 10.1038/bjc.2018.7 29485981 PMC5886128

[9] Nøst TH, Alcala K, Urbarova I, Byrne KS, Guida F, Sandanger TM, et al. Systemic inflammation markers and cancer incidence in the UK Biobank. Eur J Epidemiol. 2021;36(8):841–8. doi: 10.1007/s10654-021-00752-6 34036468 PMC8416852

[10] Hu L, Chen C. BMSCs-EVs alleviate pelvic floor dysfunction in mice by reducing inflammation and promoting tissue regeneration. In Vivo. 2024;38(6):2680–7. doi: 10.21873/invivo.13745 39477385 PMC11535908

[11] Wei B, Zhao Y, Lin P, Qiu W, Wang S, Gu C, et al. The association between overactive bladder and systemic immunity-inflammation index: a cross-sectional study of NHANES 2005 to 2018. Sci Rep. 2024;14(1):12579. doi: 10.1038/s41598-024-63448-3 38822015 PMC11143340

[12] Cao S, Meng L, Lin L, Hu X, Li X. The association between the metabolic score for insulin resistance (METS-IR) index and urinary incontinence in the United States: results from the National Health and Nutrition Examination Survey (NHANES) 2001-2018. Diabetol Metab Syndr. 2023;15(1):248. doi: 10.1186/s13098-023-01226-3 38041100 PMC10693039

[13] Yao X, Jiang M, Dong Y, Wen J, Jiang H. Association between exposure to multiple metals and stress urinary incontinence in women: a mixture approach. Environ Geochem Health. 2024;46(5):149. doi: 10.1007/s10653-024-01929-0 38578493

[14] Liu X, Ji W, Chang Y, Li Y, Li W, Cui J. Associations of life’s essential 8 with low muscle mass mediated by testosterone, inflammation, and nutritional status in United States adults: a cross-sectional study. Am J Clin Nutr. 2025;121(2):436–44. doi: 10.1016/j.ajcnut.2024.11.026 39615595

[15] Shi R, Tian Y, Tian J, Liu Q, Zhang J, Zhang Z, et al. Association between the systemic immunity-inflammation index and stroke: a population-based study from NHANES (2015-2020). Sci Rep. 2025;15(1):381. doi: 10.1038/s41598-024-83073-4 39747980 PMC11696299

[16] Chen J-H, Zhai E-T, Yuan Y-J, Wu K-M, Xu J-B, Peng J-J, et al. Systemic immune-inflammation index for predicting prognosis of colorectal cancer. World J Gastroenterol. 2017;23(34):6261–72. doi: 10.3748/wjg.v23.i34.6261 28974892 PMC5603492

[17] Guo W, Song Y, Sun Y, Du H, Cai Y, You Q, et al. Systemic immune-inflammation index is associated with diabetic kidney disease in Type 2 diabetes mellitus patients: evidence from NHANES 2011-2018. Front Endocrinol (Lausanne). 2022;13:1071465. doi: 10.3389/fendo.2022.1071465 36561561 PMC9763451

[18] Lin K-B, Fan F-H, Cai M-Q, Yu Y, Fu C-L, Ding L-Y, et al. Systemic immune inflammation index and system inflammation response index are potential biomarkers of atrial fibrillation among the patients presenting with ischemic stroke. Eur J Med Res. 2022;27(1):106. doi: 10.1186/s40001-022-00733-9 35780134 PMC9250264

[19] Lee JA, Johns TS, Melamed ML, Tellechea L, Laudano M, Stern JM, et al. Associations between socioeconomic status and urge urinary incontinence: an analysis of NHANES 2005 to 2016. J Urol. 2020;203(2):379–84. doi: 10.1097/JU.0000000000000542 31518201

[20] Delancey JOL, Ashton-Miller JA. Pathophysiology of adult urinary incontinence. Gastroenterology. 2004;126(1 Suppl 1):S23–32. doi: 10.1053/j.gastro.2003.10.080 14978635

[21] De La Torre P, Pérez-Lorenzo MJ, Alcázar-Garrido Á, Collado J, Martínez-López M, Forcén L, et al. Perinatal mesenchymal stromal cells of the human decidua restore continence in rats with stress urinary incontinence induced by simulated birth trauma and regulate senescence of fibroblasts from women with stress urinary incontinence. Front Cell Dev Biol. 2023;10:1033080. doi: 10.3389/fcell.2022.1033080 36742196 PMC9893794

[22] Wu JM. Stress incontinence in women. N Engl J Med. 2021;384(25):2428–36. doi: 10.1056/NEJMcp1914037 34161707

[23] Gacci M, Sakalis VI, Karavitakis M, Cornu J-N, Gratzke C, Herrmann TRW, et al. European Association of Urology Guidelines on male urinary incontinence. Eur Urol. 2022;82(4):387–98. doi: 10.1016/j.eururo.2022.05.012 35697561

[24] Börgermann C, Kaufmann A, Sperling H, Stöhrer M, Rübben H. The treatment of stress incontinence in men: part 2 of a series of articles on incontinence. Dtsch Arztebl Int. 2010;107(27):484–91. doi: 10.3238/arztebl.2010.0484 20661415 PMC2908931

[25] Hu JS, Pierre EF. Urinary incontinence in women: evaluation and management. Am Fam Physician. 2019;100(6):339–48. 31524367

[26] Bump RC, McClish DM. Cigarette smoking and pure genuine stress incontinence of urine: a comparison of risk factors and determinants between smokers and nonsmokers. Am J Obstet Gynecol. 1994;170(2):579–82. doi: 10.1016/s0002-9378(94)70231-4 8116716

[27] Cao S, Meng L, Lin L, Hu X, Li X. The association between the metabolic score for insulin resistance (METS-IR) index and urinary incontinence in the United States: results from the National Health and Nutrition Examination Survey (NHANES) 2001-2018. Diabetol Metab Syndr. 2023;15(1):248. doi: 10.1186/s13098-023-01226-3 38041100 PMC10693039

[28] Chen T, Zhan X, Xiao S, Fu B. U-shaped association between sleep duration and urgency urinary incontinence in women: a cross-sectional study. World J Urol. 2023;41(9):2429–35. doi: 10.1007/s00345-023-04537-2 37522906

[29] Wu S, Wu F. Association of urinary incontinence with depression among men: a cross-sectional study. BMC Public Health. 2023;23(1):944. doi: 10.1186/s12889-023-15961-9 37231365 PMC10210416

[30] Xia S, Li S, Li H. HPV-infection status and urinary incontinence: a population-based analysis of the NHANES 2005-2016. World J Urol. 2023;41(6):1597–603. doi: 10.1007/s00345-023-04425-9 37198518

[31] Xu L, Sima Y, Xiao C, Chen Y. Exosomes derived from mesenchymal stromal cells: a promising treatment for pelvic floor dysfunction. Hum Cell. 2023;36(3):937–49. doi: 10.1007/s13577-023-00887-6 36940057

[32] Alexandre EC, Calmasini FB, Sponton ACDS, de Oliveira MG, André DM, Silva FH, et al. Influence of the periprostatic adipose tissue in obesity-associated mouse urethral dysfunction and oxidative stress: effect of resveratrol treatment. Eur J Pharmacol. 2018;836:25–33. doi: 10.1016/j.ejphar.2018.08.010 30102890

[33] Gill K, Horsley H, Swamy S, Khasriya R, Malone-Lee J. A prospective observational study of urinary cytokines and inflammatory response in patients with Overactive Bladder Syndrome. BMC Urol. 2021;21(1):39. doi: 10.1186/s12894-021-00809-4 33740940 PMC7980577

[34] Post WM, Widomska J, Grens H, Coenen MJH, Martens FMJ, Janssen DAW, et al. Molecular processes in stress urinary incontinence: a systematic review of human and animal studies. Int J Mol Sci. 2022;23(6):3401. doi: 10.3390/ijms23063401 35328824 PMC8949972

[35] Tang J, Liu C, Min J, Hu M, Li Y, Hong L. Potential therapeutic role of punicalagin against mechanical-trauma-induced stress urinary incontinence via upregulation of Nrf2 and TGF-β1 signaling : Effect of punicalagin on mechanical trauma induced SUI. Int Urogynecol J. 2017;28(6):947–55. doi: 10.1007/s00192-017-3283-x 28168411 PMC5437194

[36] Li Y, Li B-S, Liu C, Hong S-S, Min J, Hu M, et al. Effect of integrin β1 in the treatment of stress urinary incontinence by electrical stimulation. Mol Med Rep. 2019;19(6):4727–34. doi: 10.3892/mmr.2019.10145 31059065 PMC6522829

[37] Lo T-S, Lin Y-H, Uy-Patrimonio MC, Chu H-C, Hsieh W-C, Chua S. Dissecting of the paravesical space associated with lower urinary tract dysfunction - a rat model. Sci Rep. 2020;10(1):1718. doi: 10.1038/s41598-020-58604-4 32015355 PMC6997187

[38] Koch M, Umek W, Hanzal E, Mohr T, Seyfert S, Koelbl H, et al. Serum proteomic pattern in female stress urinary incontinence. Electrophoresis. 2018;39(8):1071–8. doi: 10.1002/elps.201700423 29359342

[39] Heinrich M, Oberbach A, Schlichting N, Stolzenburg J-U, Neuhaus J. Cytokine effects on gap junction communication and connexin expression in human bladder smooth muscle cells and suburothelial myofibroblasts. PLoS One. 2011;6(6):e20792. doi: 10.1371/journal.pone.0020792 21674053 PMC3107230

[40] Wang Z, Cheng Z, Cristofaro V, Li J, Xiao X, Gomez P, et al. Inhibition of TNF-α improves the bladder dysfunction that is associated with type 2 diabetes. Diabetes. 2012;61(8):2134–45. doi: 10.2337/db11-1763 22688336 PMC3402324

[41] Borrego-Jimenez P-S, Flores-Fraile J, Padilla-Fernández B-Y, Valverde-Martinez S, Gómez-Prieto A, Márquez-Sánchez MT, et al. Improvement in quality of life with pelvic floor muscle training and biofeedback in patients with painful bladder syndrome/interstitial cystitis. J Clin Med. 2021;10(4):862. doi: 10.3390/jcm10040862 33669734 PMC7922867

[42] Jhang J-F, Kuo H-C. Novel treatment of chronic bladder pain syndrome and other pelvic pain disorders by OnabotulinumtoxinA injection. Toxins (Basel). 2015;7(6):2232–50. doi: 10.3390/toxins7062232 26094697 PMC4488700

[43] Murphy AB, Macejko A, Taylor A, Nadler RB. Chronic prostatitis: management strategies. Drugs. 2009;69(1):71–84. doi: 10.2165/00003495-200969010-00005 19192937

[44] Tan Q, Le H, Tang C, Zhang M, Yang W, Hong Y, et al. Tailor-made natural and synthetic grafts for precise urethral reconstruction. J Nanobiotechnology. 2022;20(1):392. doi: 10.1186/s12951-022-01599-z 36045428 PMC9429763

[45] Yoshimura N, Oguchi T, Yokoyama H, Funahashi Y, Yoshikawa S, Sugino Y, et al. Bladder afferent hyperexcitability in bladder pain syndrome/interstitial cystitis. Int J Urol. 2014;21 Suppl 1(0 1):18–25. doi: 10.1111/iju.12308 24807488 PMC4089034

[46] Chess-Williams R, McDermott C, Sellers DJ, West EG, Mills KA. Chronic psychological stress and lower urinary tract symptoms. Low Urin Tract Symptoms. 2021;13(4):414–24. doi: 10.1111/luts.12395 34132480

[47] Lloyd GL, Marks JM, Ricke WA. Benign prostatic hyperplasia and lower urinary tract symptoms: what is the role and significance of inflammation? Curr Urol Rep. 2019;20(9):54. doi: 10.1007/s11934-019-0917-1 31377881 PMC7339114

[48] Hsiao S-M, Lin H-H, Kuo H-C. The role of serum C-reactive protein in women with lower urinary tract symptoms. Int Urogynecol J. 2012;23(7):935–40. doi: 10.1007/s00192-012-1715-1 22422219

[49] Paz-Levy D, Weintraub AY, Reuven Y, Yohay Z, Idan I, Elharar D, et al. Prevalence and risk factors for urinary tract infection following stress urinary incontinence surgery with two midurethral sling procedures. Int J Gynaecol Obstet. 2018;143(3):333–8. doi: 10.1002/ijgo.12680 30229894

